# Supplementary material for: Causal Relationship between Aging and Anorexia Nervosa: A White-Matter-Microstructure-Mediated Mendelian Randomization Analysis
Source: Biomedicines. 2024 Aug 16;12(8):1874. doi: 10.3390/biomedicines12081874 (PMC11351342; doi:10.3390/biomedicines12081874)
Supplement: Supplementary file 1 [file biomedicines-12-01874-s001.zip › Supplementary Figure/Figure S8/Leaveoneout plot of MR analysis for TL to ubm-b-1978.pdf]

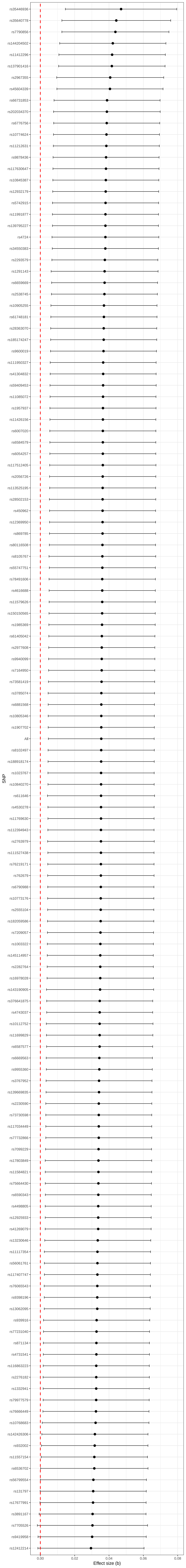
rs35446936
rs35640778
rs7790856
rs144204502
rs11412296
rs137901416
rs2967355
rs45604339
rs66731853
rs202034370
rs6776756
rs10774624
rs11212631
rs9878436
rs117630647
rs10845387
rs12932179
rs5742915
rs11991877
rs139795227
rs4724
rs34550383
rs2293579
rs1291143
rs6659669
rs2538745
rs10905255
rs61748181
rs28363070
rs185174247
rs9600019
rs111950327
rs41304832
rs59409453
rs11085072
rs1957937
rs11426156
rs6007020
rs6584579
rs6054257
rs117512405
rs2056726
rs113525195
rs28502153
rs450962
rs12369950
rs869785
rs80116508
rs8105767
rs55747751
rs78491606
rs4616688
rs11579626
rs150150565
rs1985369
rs61405042
rs2977608
rs9940099
rs7164950
rs73581419
rs3785074
rs6881568
rs10805346
rs1907702
A8
rs8102497
rs188918174
rs1023767
rs10840270
rs611646
rs4530278
rs11769630
rs112394943
rs2763979
rs111527438
rs76219171
rs762679
rs6790988
rs10773176
rs2555104
rs182059586
rs7209057
rs1003322
rs145114957
rs2282764
rs16978028
rs143190905
rs376641875
rs4743037
rs10112752
rs11699829
rs6587577
rs6669563
rs9955360
rs3767952
rs139669835
rs2230590
rs73730598
rs117034449
rs77732866
rs7099229
rs17803849
rs11584821
rs75664430
rs6590343
rs4498805
rs12925933
rs41269079
rs13230646
rs11117354
rs56061761
rs117407747
rs76065543
rs9398196
rs13062095
rs939916
rs77231040
rs871134
rs4731541
rs116863223
rs2276182
rs1332941
rs79977579
rs76666449
rs10768683
rs142426306
rs932002
rs11557154
rs6536702
rs56799554
rs131797
rs17677991
rs3891167
rs7705526
rs9419958
rs12412214
SNP
0.00
0.02
0.04
0.06
0.08
Effect size (b)
